# Exposure to 2-amino-1-methyl-6-phenylimidazo[4,5-b]pyridine causes cecal microbiota dysbiosis in mice

**DOI:** 10.3389/fmicb.2026.1859419

**Published:** 2026-06-15

**Authors:** Nesreen Aljahdali

**Affiliations:** 1 Department of Biological Science, Faculty of Science, King Abdulaziz University, Jeddah, Saudi Arabia; 2 Stem Cells Research Unit, King Fahd Medical Research Center, King Abdulaziz University, Jeddah, Saudi Arabia

**Keywords:** cecal microbiota, dietary carcinogens, heterocyclic amines, Maillard reaction products, PhIP and gut microbiota

## Abstract

2-Amino-1-methyl-6-phenylimidazo[4,5-b]pyridine (PhIP) is a heterocyclic amine (HCA) that is formed during high-temperature meat cooking, especially grilling, and is classified as a carcinogenic food-borne compound that can impact multiple physiological systems. The aim of this study was to evaluate how exposure to PhIP affects the composition of the cecal microbiota in mice. A total of 32 mice were randomly divided into four groups: A negative control group, a positive control group, and two groups treated orally with either 10 or 20 mg/kg PhIP for 2 months. The V3-V4 regions of the bacterial 16S rRNA gene were sequenced to characterize the cecal microbial communities. Microbial richness and diversity decreased in the PhIP-treated mice compared to the negative control group. At the phylum level, the relative abundance of Bacillota and Actinomycetota decreased significantly after PhIP exposure, while the abundance of Campylobacterota and Bacteroidota increased. At the genus level, the abundance of *Roseburia*, *Lactobacillus*, and *Bifidobacterium* decreased, while the abundance of *Blautia*, *Lachnospiraceae*-NK4A136 group, *Bacteroides*, *Alistipes*, *Helicobacter*, and *Escherichia*–*Shigella* increased. The results highlight that bacterial community composition and its relative abundance in the cecum change with PhIP exposure and offer further evidence of microbiota dysbiosis that occurs with PhIP exposure.

## 1 Introduction

The non-enzymatic browning reaction between reducing sugars and free amino groups, which leads to the formation of Maillard reaction products (MRPs), is one of the key chemical transformations in foods. This reaction was first described by Louis Camille Maillard in 1912 during studies on heat-induced changes in amino acid–sugar systems (Gupta et al., 2018). MRPs are widely present in Western dietary patterns, occurring in thermally processed foods such as bread, baked potato, cake, roast pork, breakfast cereal, and pastry. These compounds play a pivotal role in the formation and modulation of the flavor, color, and aroma characteristics of foods during heat treatment (Tuohy et al., 2006). The formation and progression of MRPs can be modulated by multiple physicochemical parameters, including temperature, pH, and water activity (Ames, 1990). These factors can lead to changes in the reaction paths, rate, and end products (Sumaya-Martinez et al., 2005). The progression of RPs can be divided into three principal phases: The initial phase, the intermediate phase, and the final phase (Hodge, 1953). Each stage of food preparation, including the application of various cooking techniques, induces a series of complex chemical reactions that lead to the formation of numerous process-induced compounds, such as Nϵ-fructosyllysine (furosine), 5-hydroxymethylfurfural (HMF), acrylamide, heterocyclic amines (HCAs), advanced glycation end products (AGEs), and melanoidins (ALjahdali and Carbonero, 2019). HCAs, which are formed through reactions among reducing sugars, amino acids, and their precursor creatine, are produced during the cooking of skeletal muscle meats, including beef, pork, poultry, and fish (Jägerstad et al., 1991; Tuohy et al., 2006). Evidence has shown that the formation of HCAs in meat occurs due to increases in temperature above 200 °C or when meat is cooked for prolonged periods at lower temperatures (Jägerstad et al., 1991, Tuohy et al., 2006). The formation of HCAs in thermally processed meat is influenced by both the type of meat and the specific cooking conditions employed. For example, beef cooked to a well-done degree exhibits substantially higher concentrations of HCAs compared to beef cooked to a medium degree of doneness (Puangsombat et al., 2012). The levels of HCAs in different types of meat have been quantified. Specifically, it has been reported that the concentrations of HCAs in fried bacon, fried pork, fried beef, and fried chicken are 17.59 ng/g, 13.91 ng/g, 8.92 ng/g, and 7.06 ng/g, respectively (Puangsombat et al., 2012). Several types of HCAs have been identified and investigated in food matrices, including 2-amino-3-methylimidazo [4,5-f]quinoline (IQ), 2-amino-3,4-dimethylimidazo [4,5-f]quinoline (MeIQ), 2-amino-3,8-dimethylimidazo[4,5-f]quinoxaline (MeIQx), and 2-amino-1-methyl-6-phenylimidazo[4,5-b]pyridine (PhIP). These compounds are classified as carcinogenic by the International Agency for Research on Cancer (IARC Working Group on the Evaluation of Carcinogenic Risks to Humans, International Agency for Research on Cancer, and World Health Organization, 1993).

PhIP was first discovered in fried ground beef cooked at 300 °C (Felton et al., 1986). It has been reported that the concentration of PhIP in cooked beef reached 182 ng/g, whereas in cooked chicken it was 49 ng/g (Augustsson et al., 1997; Felton et al., 2002; Sugimura et al., 2004). Furthermore, it was estimated that the amount of PhIP was 5.22 ng/g in fried camel meat (Khan et al., 2017; Rizwan Khan et al., 2017). Several studies have demonstrated that PhIP is absorbed through the intestinal tract and subsequently excreted in urine as metabolites within 24 h following the ingestion of barbecued meat (Kulp et al., 2004; Lynch et al., 1992). Numerous epidemiological investigations have demonstrated an association between dietary exposure to PhIP and adverse health outcomes. For example, previous studies have reported that PhIP induces intestinal carcinomas and lymphomas in both male and female rats (Ochiai et al., 2002), as well as mammary carcinomas in female rats (Imaida et al., 1996). In addition, it has been reported that PhIP is detectable in human breast milk and can be transmitted from the mother to the offspring via lactational exposure (Jägerstad et al., 1994). Moreover, studies have found that PhIP could cause prostate and colon carcinoma in rodents (Li et al., 2012; Nicken et al., 2013). Acute exposure to PhIP within the colonic environment may induce oxidative stress, as evidenced by elevated nitrotyrosine concentrations and a concomitant reduction in the enzymatic activities of glutathione peroxidase (GSH-Px) and superoxide dismutase (SOD) (Chen et al., 2017; Li et al., 2013). Moreover, short-term exposure to PhIP was found to disrupt colonic energy metabolism, characterized by an upregulation of tricarboxylic acid cycle activity and a concomitant inhibition of glycolysis (Zhang et al., 2024).

It is now widely recognized that the gastrointestinal tract (GIT) harbors the majority of the gut-associated microbial community (Ley et al., 2006). The bacterial population densities in the small and large intestines are approximately 10^5^–10^6^ cells/mL and 10^8^–10^9^ cells/mL, respectively. These bacteria produce short-chain fatty acids (SCFAs) through the fermentation and degradation of dietary substrates. In addition to bacteria, other components of the gut microbiota are present in the large intestine, including Archaea, protozoa, viruses, and fungi; however, the gut microbiota is predominantly composed of anaerobic bacterial species (Walter and Ley, 2011). The interaction between dietary factors and the gut microbiota has been implicated in both the pathogenesis and prevention of various diseases (Louis et al., 2007). The possibility that the intestinal microbiota convert PhIP into additional toxic metabolites must be considered. A previous study demonstrated that, upon incubation with human stool samples, PhIP was biotransformed into 7-hydroxy-5-methyl-3-phenyl-6,7,8,9-tetrahydropyrido[3′,2′:4,5]-imidazo[1,2-a]pyrimidin-5-ium chloride (PhIP-M1) by strains belonging to the species *Enterococcus faecium, Enterococcus durans, Enterococcus avium,* and *Lactobacillus reuteri* (Vanhaecke et al., 2006; Vanhaecke et al., 2007). With respect to PhIP-M1 toxicity, it has been demonstrated that exposure to 100–200 μM PhIP-M1 induces DNA damage, apoptosis, and cell cycle arrest in Caco-2 cells (Vanhaecke et al., 2008). However, an additional study indicated that this concentration of PhIP-M1 is unlikely to exert cytotoxic or carcinogenic effects on the colorectal mucosa (Nicken et al., 2015). Previous studies have demonstrated that exposure of rat fecal samples to PhIP resulted in an increased relative abundance of the bacterial family Muribaculaceae, concomitant with decreased relative abundances of the families Ruminococcaceae and Lactobacillaceae (Zhao et al., 2021). A recent study demonstrated that co-administration of PhIP and dextran sulfate sodium (DSS) resulted in an increased relative abundance of the bacterial taxon *Clostridia* UCG014 in rat fecal samples (Zapico et al., 2024). The impact of PhIP on biological systems, and in particular on the composition of the cecal microbiota, remains poorly characterized. Consequently, the objective of this study was to elucidate the effects of PhIP exposure on cecal microbiota composition in mice.

## 2 Materials and methods

### 2.1 Chemicals

PhIP (purity: 98.9%) and corn oil were obtained from Aladdin Scientific (California, United States).

### 2.2 Experimental animals

The animal experiment was conducted at the Animal House Facility, Faculty of Pharmacy, King Abdulaziz University. A total of 32 male *BALB/c* mice, 8 weeks of age and with a body weight of 20 g, were randomly allocated to four experimental groups (n = 8 per group). The animals were housed in groups per cage and maintained under a 12 h light/12 h dark photoperiod at standard room temperature (20 ± 2 °C) and relative humidity (50 ± 5%), with ad libitum access to standard chow and water.

### 2.3 Experimental design

**Figure 1:**
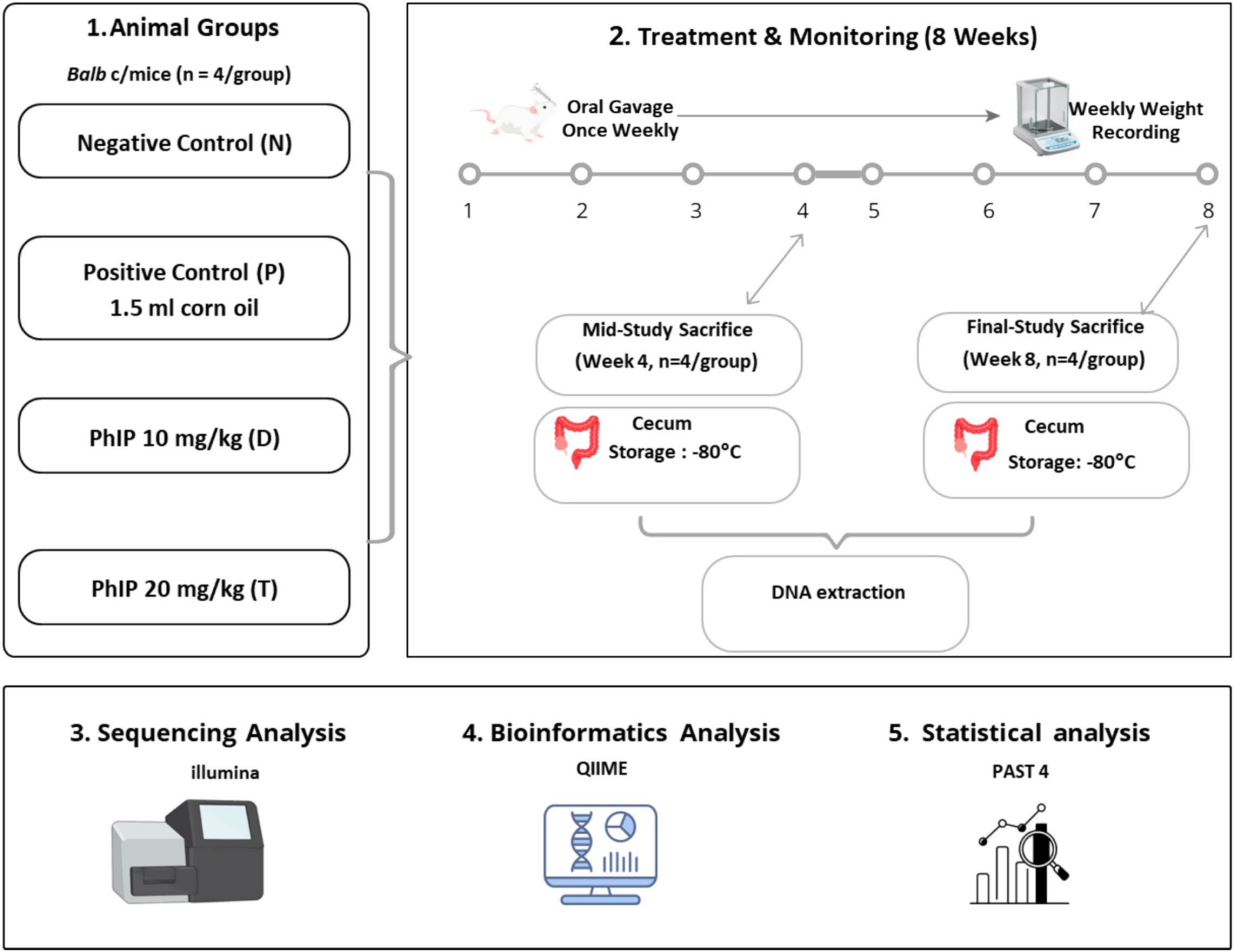
Overview of the study design and procedures for data collection and analysis.

A total of 32 mice were randomly assigned to four experimental groups and housed in separate cages, with eight animals per group. Group 1 served as the negative control (N) and received distilled water; this group was further subdivided into N1 and N2, corresponding to animals sacrificed after the first and second months, respectively. Group 2 served as the positive control (P) and received 1.5 mL of corn oil; this group was similarly subdivided into P1 and P2 for the first and second months, respectively. Group 3 received PhIP at a dose of 10 mg/kg body weight and was designated as group D, with subgroups M1D and M2D representing animals sampled at the end of the first and second months, respectively. Group 4 received PhIP at a dose of 20 mg/kg body weight and was designated as group T, with subgroups M1T and M2T corresponding to the first and second months, respectively (Li et al., 2013, Kimura et al., 2003). Mice in the treatment groups were administered PhIP by oral gavage for a total duration of 2 months. For oral administration, PhIP was dissolved in corn oil at a dosing volume of 10 mL/kg body weight, in accordance with previously described protocols (Diehl et al., 2001). At the end of the experimental period, mice were euthanized and subjected to necropsy. Cecal contents were collected immediately under anaerobic conditions for downstream sequencing and microbiota analyses (Figure 1).

### 2.4 DNA extraction and sequencing

Genomic DNA was extracted from the cecal samples using the commercial QIAamp DNA Stool and Tissue Mini Kit (Qiagen, Germany) according to the manufacturer’s instructions. Target regions were amplified by PCR using specific primers conjugated to sample-specific barcodes, followed by agarose gel electrophoresis to select amplicons of the desired size. The PCR amplification targeted the V3-4 hypervariable regions of the bacterial 16S rRNA gene. Sequencing libraries were prepared using the Nextera XT DNA Library Preparation Kit (Illumina) and sequenced on an Illumina MiSeq platform in a 2 × 250 bp paired-end configuration, following the manufacturer’s protocol.

### 2.5 Bioinformatics and statistical analyses

From 32 samples, a total of 12,877,243 raw sequence reads were obtained, of which 10,089,340 high-quality reads were retained for downstream analyses. FASTQ files were processed using the Quantitative Insights into Microbial Ecology (QIIME) pipeline, which enables comprehensive microbial community analysis (Kuczynski et al., 2012). Briefly, raw reads were merged and quality filtered to generate clean sequences. Representative sequences of each amplicon sequence variant (ASV) were subsequently taxonomically annotated to determine the corresponding species identities and their relative abundances. ASV tables were used to assess species richness, calculate α-diversity indices, and construct Venn and flower diagrams. To evaluate differences in microbial community composition and structure between the groups, Student’s *t*-tests and MetagenomeSeq analyses were performed. In addition, nonparametric comparisons were conducted using the Kruskal–Wallis test followed by Dunn’s *post hoc* tests in PAST software (version 4.03). Microbiota data were ordinated using non-metric multidimensional scaling (NMDS) based on Bray–Curtis similarity. The statistical significance of subgroup clustering was assessed using permutational multivariate analysis of variance (PERMANOVA), with a significance threshold set at a *p*-value of < 0.05.

## 3 Results

### 3.1 Operational taxonomic unit analysis

#### 3.1.1 Venn diagram

**Figure 2:**
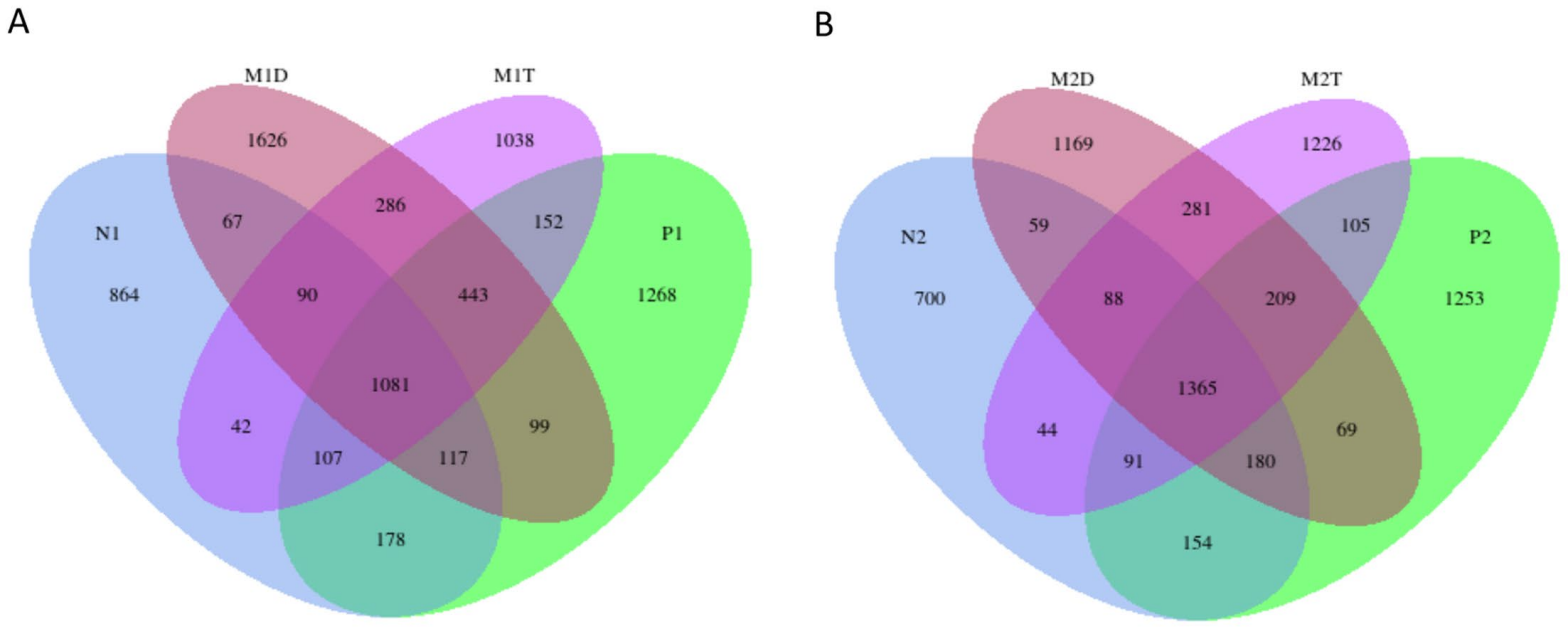
Venn diagram representing the total number of OTUs across all groups. **(A)** Different colors indicate different groups in the first month. **(B)** Different colors indicate different groups in the second month. The overlapping circles represent shared OTUs between groups.

A Venn diagram was used to depict the shared core taxa among the different microbial community groups. In the first month, a total of 7,458 OTUs were identified, of which 1,081 OTUs were shared across all groups. In the second month, a total of 6,993 OTUs were detected, with 1,365 OTUs shared by all groups. In the first month, the number of OTUs in the negative control group (N1) was 864, whereas the positive control group (P1) harbored 1,268 OTUs. The treated groups M1D and M1T contained 1,626 and 1,038 OTUs, respectively. In the second month, the negative control group (N2) comprised 700 OTUs, while the positive control group (P2) comprised 1,253 OTUs. The treated groups M2D and M2T contained 1,169 and 1,226 OTUs, respectively (Figures 2A,B).

#### 3.1.2 Alpha diversity analysis and rarefaction curves

**Figure 3:**
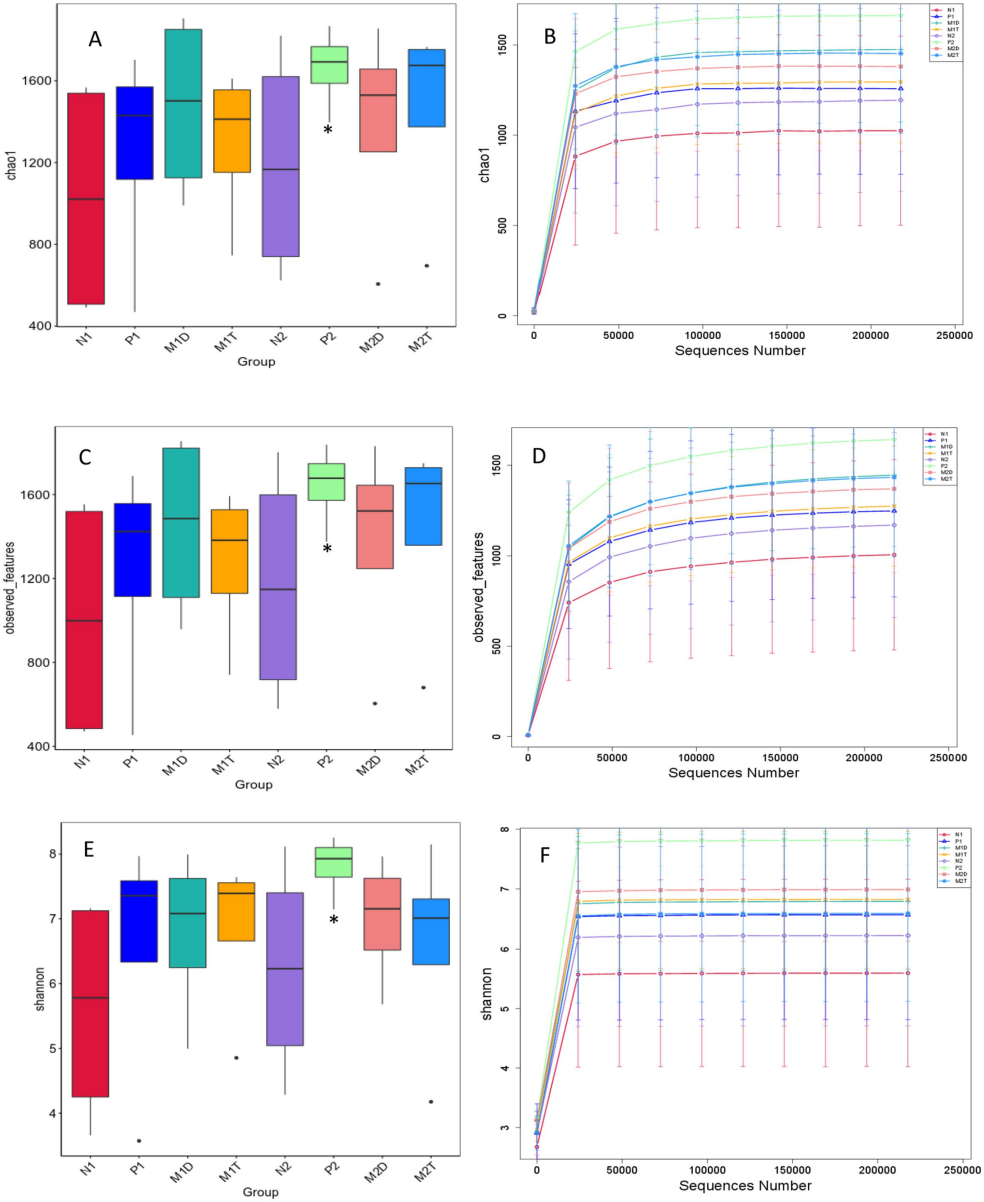
Alpha diversity metrics calculated from OTUs using the Chao1, observed species, and Shannon indices. **(A,C,E)** Boxplots comparing species richness and community distribution among all groups. The lines within the boxes represent the median, and the box boundaries represent the 25th and 75th percentiles, respectively. **(B,D,F)** Rarefaction curves showing the observed number of species across all groups. The abscissa indicates the number of sequences, and the ordinate indicates the average number of OTUs per sample in each group.

**Figure 4:**
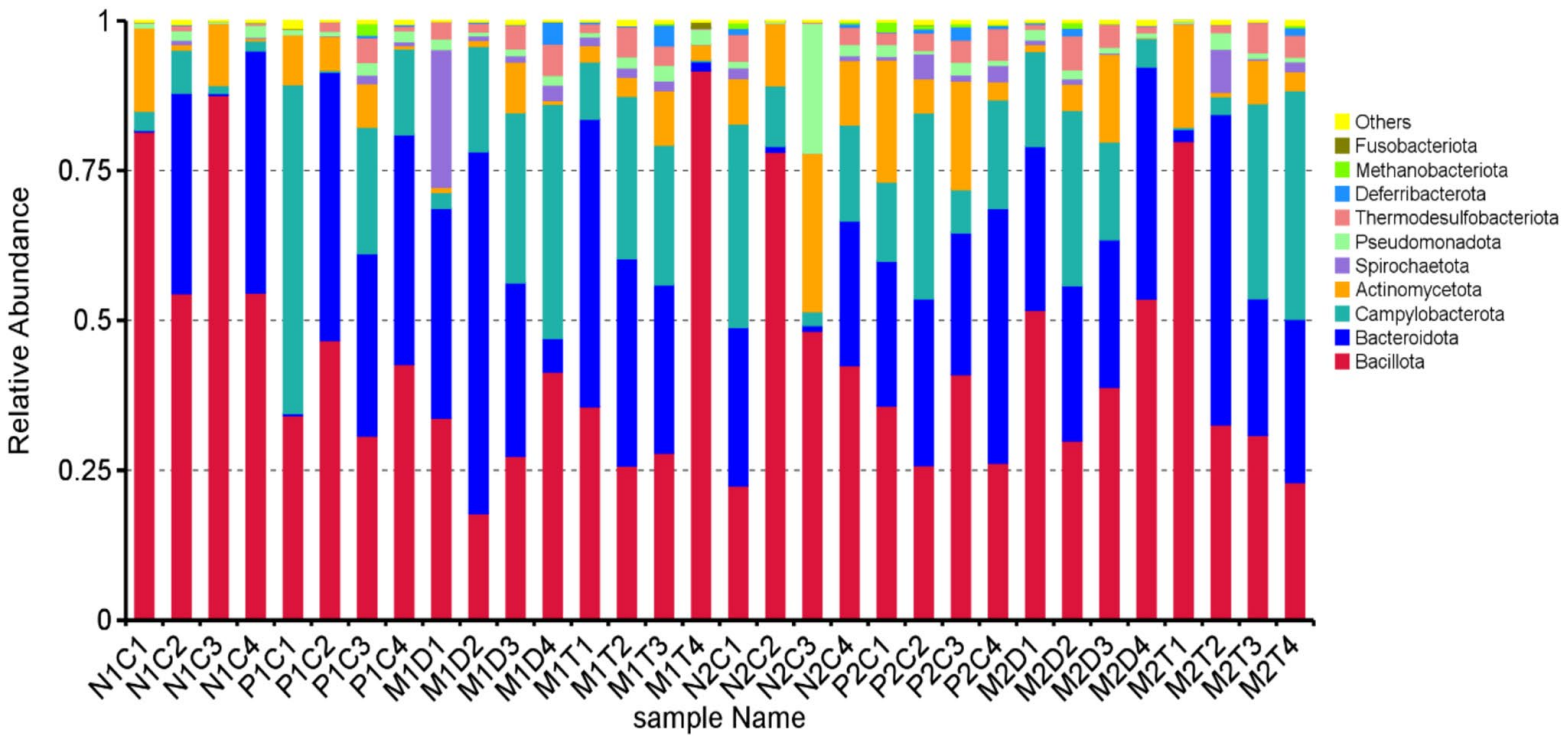
Relative abundance within a single sample. Higher diversity and richness of phyla were observed in the negative control groups (N1C and N2C) compared to the positive control (P1C and P2C) and the treated groups (M1D, M2D, M1T, and M2T).

The diversity within a microbial community is called alpha diversity. Within-sample diversity was assessed using the Chao1, observed species, and Shannon indices in the present study. These indices are used to estimate the number and diversity of species, including rare species that are not always observed in one sample. Alpha diversity was not significantly different among the four experimental groups in the first month. However, the negative control group (N1) showed a tendency toward greater richness and diversity than the other groups. The same situation was seen in the second month, with no overall significant differences between the four groups; however, the negative control group (N2) again showed higher richness and diversity than the treated groups. Notably, there was a statistically significant difference between the negative control group (N2) and the positive control group (P2) (Kruskal–Wallis, *p* < 0.05; Figures 3A,C,E). Rarefaction curves based on the Chao1, observed species, and Shannon indices were created to assess sequencing depth and to illustrate alpha diversity for all samples. Species richness at various sequencing depths for each group is shown in these curves (Figures 3B,D,F), suggesting that the sequencing depth was sufficient to cover most of the bacterial diversity in each group. The relative abundance of bacterial phyla at the sample level was also analyzed. The relative abundance of Bacillota (formerly Firmicutes)—a phylum comprising several well-characterized probiotic taxa—was higher in the negative control group (N) than in the treated groups (Figure 4).

### 3.2 Impact of PhIP on the composition of the cecal microbiota

#### 3.2.1 Impact of PhIP on gut microbiota profiles and dynamics

**Figure 5:**
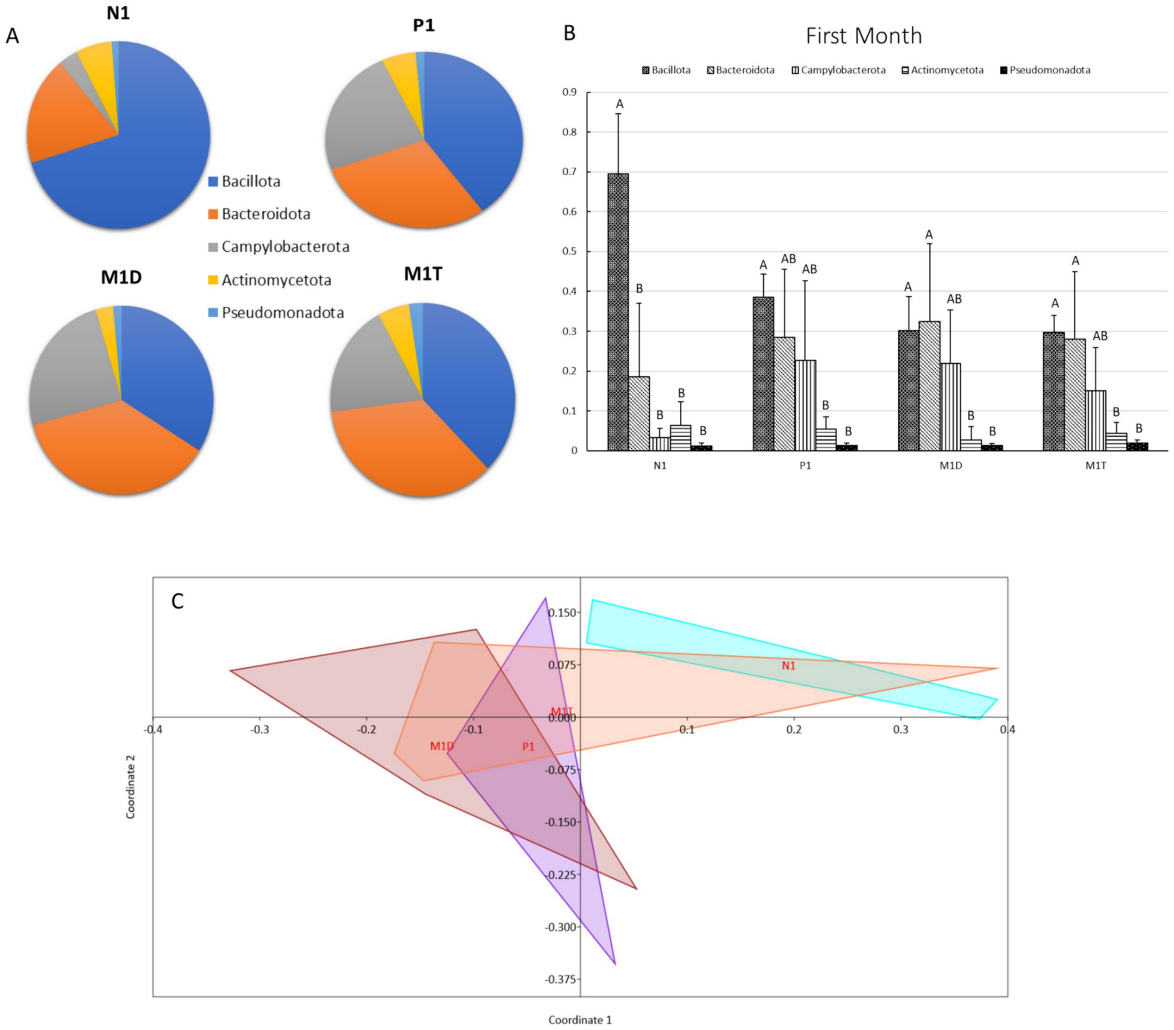
Relative abundance of Bacillota, Bacteroidota, Campylobacterota, Actinomycetota, and Pseudomonadota among the four groups in the first month. **(A)** Pie chart; **(B)** bar chart showing significant differences indicated by different letters (Kruskal–Wallis test and Dunn’s *post hoc* test, *p* < 0.05); **(C)** NMDS (Bray–Curtis similarity index): N1 (blue color), P1 (purple color), M1D (red color), and M1T (orange color). A significant difference was observed between N1 and M1D (PERMANOVA, *p* < 0.05).

In the 32 mice, the effects of PhIP on the composition of the cecal microbiota were evaluated. The pie chart (Figure 5A) shows the predominant bacterial phyla in the first month, including Bacillota (formerly Firmicutes), Bacteroidota, Campylobacterota, Actinomycetota (formerly Actinobacteria), and Pseudomonadota (formerly Proteobacteria). Bacillota was the most abundant phylum in the negative control group (N1), with a relative abundance of 0.69, while Bacteroidota, Campylobacterota, Actinomycetota, and Pseudomonadota accounted for 0.186, 0.033, 0.063, and 0.011, respectively. The relative abundance of Bacillota also significantly dropped in the positive control group (P1) to 0.38, while the abundances of Bacteroidota and Campylobacterota significantly rose to 0.28 and 0.22, respectively. The relative abundances of Actinomycetota and Pseudomonadota were relatively low at 0.054 and 0.013, respectively. The level of Bacillota further decreased in the group that was given PhIP at 10 mg/kg (M1D), while the levels of Bacteroidota and Campylobacterota moderately increased in the same group to 0.324 and 0.219, respectively. The abundance of Actinomycetota decreased to 0.027, and Pseudomonadota remained at a low abundance of 0.013. Bacillota accounted for 0.452 of the relative abundance in the 20 mg/kg PhIP-treated group (M1T), while Bacteroidota and Campylobacterota increased to 0.280 and 0.150, respectively. The relative abundances of Actinomycetota and Pseudomonadota were also low (0.043 and 0.019, respectively), as indicated in the bar chart (Figure 5B). Non-metric multidimensional scaling (NMDS) based on the Bray–Curtis similarity index showed that the microbial community structure was different among the four groups. All major phyla were found in all groups, but the samples from the negative control group (N1) were grouped more closely together than the others. The communities of N1 and M1D differed from each other significantly according to PERMANOVA (*p* < 0.05) (Figure 5C).

**Figure 6:**
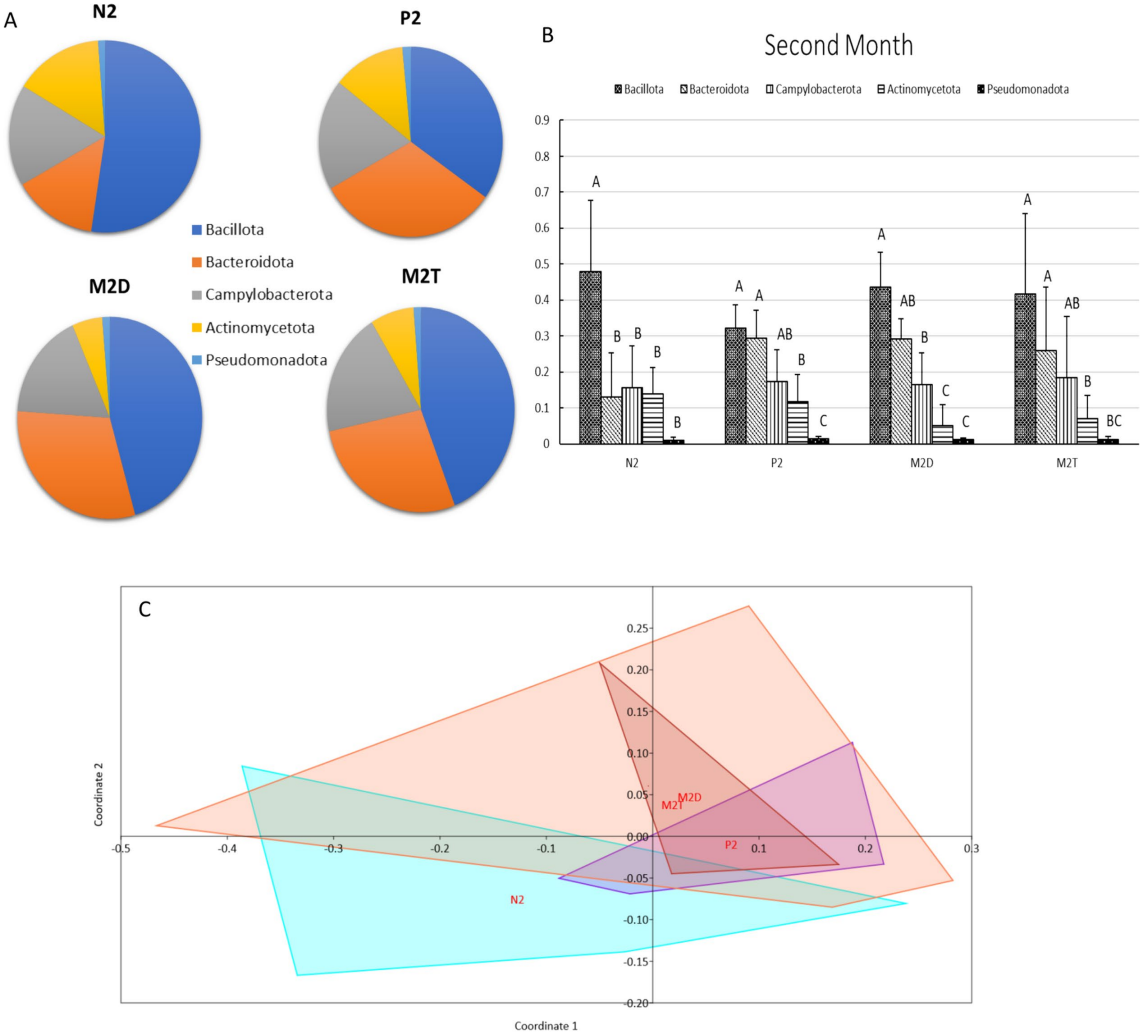
Relative abundance of Bacillota, Bacteroidota, Campylobacterota, Actinomycetota, and Pseudomonadota among the four groups in the second month. **(A)** Pie chart; **(B)** bar chart showing significant differences indicated by different letters (Kruskal–Wallis test and Dunn’s *post hoc* test, *p* < 0.05); **(C)** NMDS (Bray–Curtis similarity index): N2 (blue color), P2 (purple color), M2D (red color), and M2T (orange color). No significant difference was observed among all groups (PERMANOVA, *p* < 0.05).

The second month also showed varying data in terms of phylum-level distribution among the groups, as shown in Figure 6A. In the negative control group (N2), the dominant phylum was still Bacillota, with a relative abundance of 0.47, while the other phyla were present at relative abundances of 0.131, 0.156, 0.137, and 0.0107, respectively. The relative abundance of Bacillota decreased in the positive control group (P2) to 0.322, and the abundance of Actinomycetota also slightly decreased to 0.118. In comparison, Bacteroidota, Campylobacterota, and Pseudomonadota had moderate growth values of 0.295, 0.174, and 0.0138, respectively. In the PhIP-treated groups, Bacillota exhibited a slight decrease in both M2D (0.435) and M2T (0.416). A decrease was also observed in Actinomycetota in M2D (0.050) and M2T (0.070). Bacteroidota increased in both M2D and M2T, with a value of 0.295 and 0.291, respectively, while Campylobacterota also increased to 0.165 and 0.185, respectively. As shown in Figure 6B, the relative abundance of Pseudomonadota was low, although slightly higher in both treated groups (M2D and M2T), at 0.012 and 0.013, respectively. Furthermore, NMDS analysis based on the Bray–Curtis similarity index revealed that there was no tight grouping of samples from the negative control group (N2) with those from the treated groups. In the second month, there were no significant differences in the overall community composition among the groups (Figure 6C).

#### 3.2.2 Impact of PhIP at the phylum level

**Figure 7:**
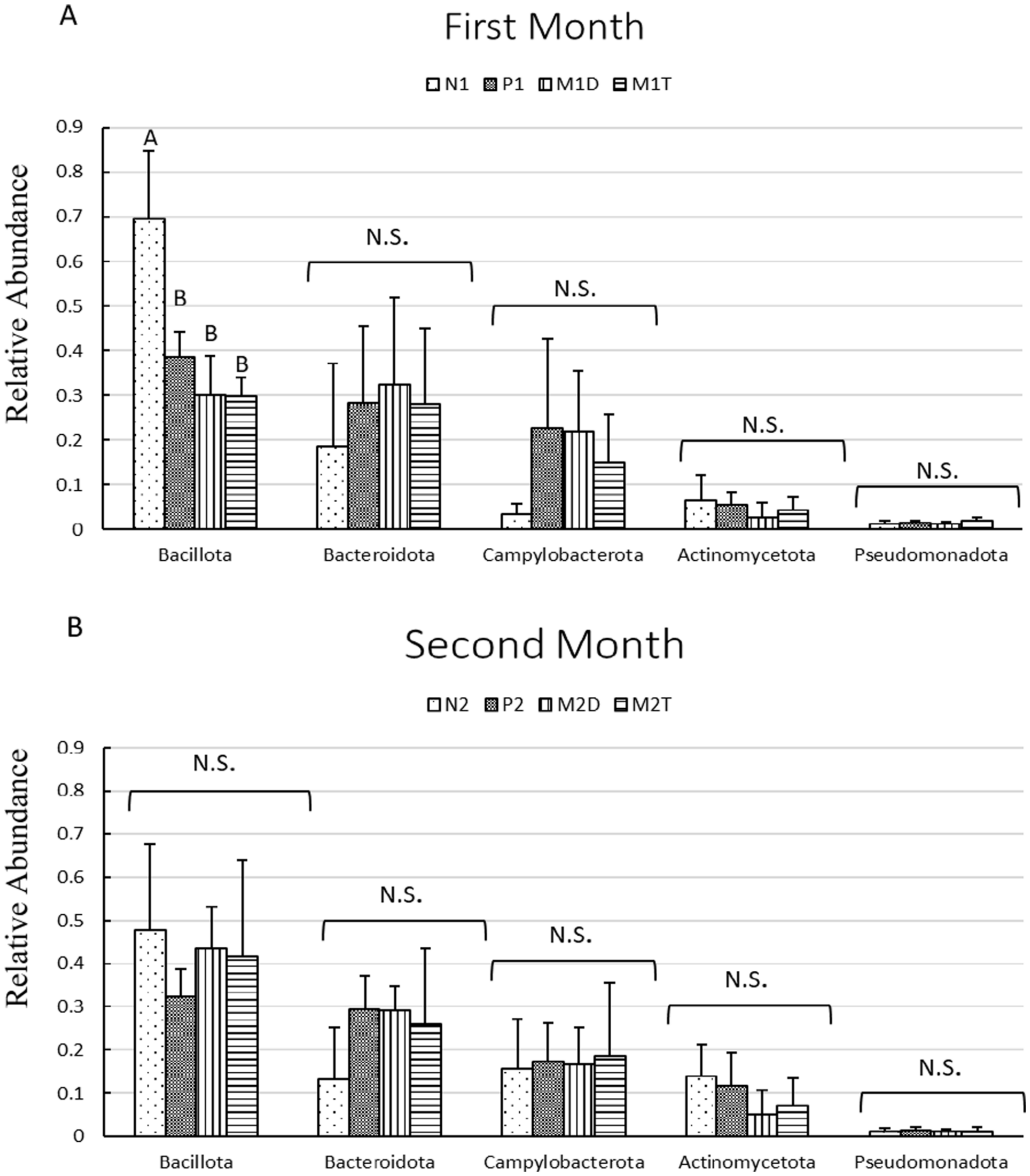
Relative abundance at the phylum level during the study: **(A)** First month; **(B)** second month. Significant differences (*p* < 0.05) are indicated by different letters. N.S. indicates no significant difference.

The relative abundance of Bacillota varied significantly between the groups in the first month, with lower abundance observed in the PhIP-treated groups. There were no differences in the abundance of Bacteroidota between the groups, but relative abundance was higher in the treated groups. Similarly, Campylobacterota did not exhibit any significant differences among the groups, but higher abundances were also observed in the treated groups. The relative abundance of Actinomycetota in the treated groups, on the other hand, decreased, although the change was not significant. Similarly, Pseudomonadota tended to be more abundant in the treated groups, with no significant difference between the groups. In the second month, the relative abundance of Bacillota remained lower in the treated groups; however, no significant differences were detected among the groups. A reduction in abundance was also noted in the treated groups for Actinomycetota, although this was not statistically significant. There was no significant difference between the groups for either Bacteroidota or Campylobacterota, but both of these phyla had relatively higher percentages in the treated groups. The relatively higher abundance of Pseudomonadota in the treated groups (Figures 7A,B) did not result in significant differences between the groups.

#### 3.2.3 Impact of PhIP at the genus level

**Figure 8:**
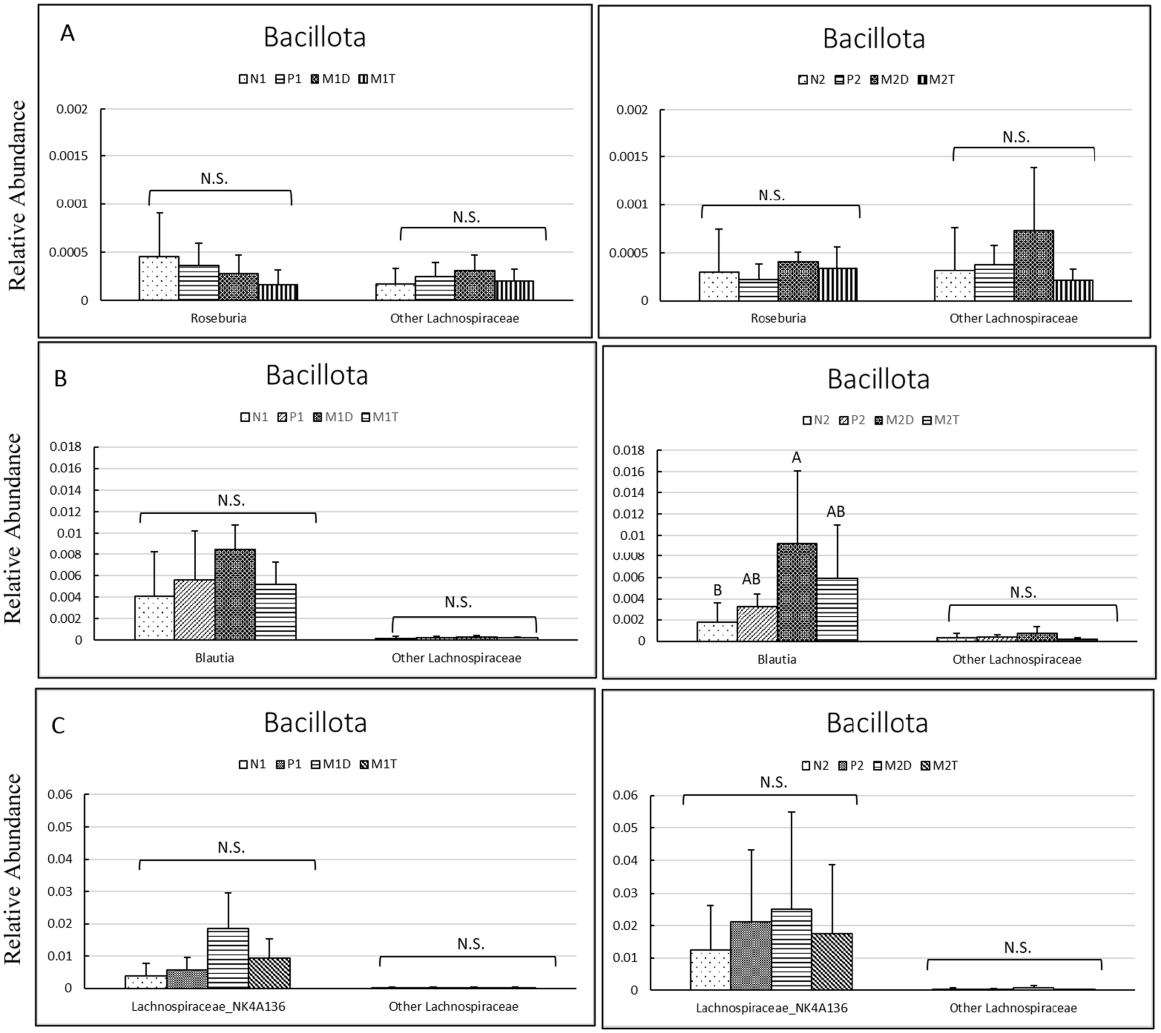
Relative abundance of Lachnospiraceae’s genera during the study. **(A)** *Roseburia*; **(B)** *Blautia*; **(C)** *Lachnospiraceae_NK4A136*. Significant differences (*p* < 0.05) are indicated by different letters. N.S. indicates no significant difference.

**Figure 9:**
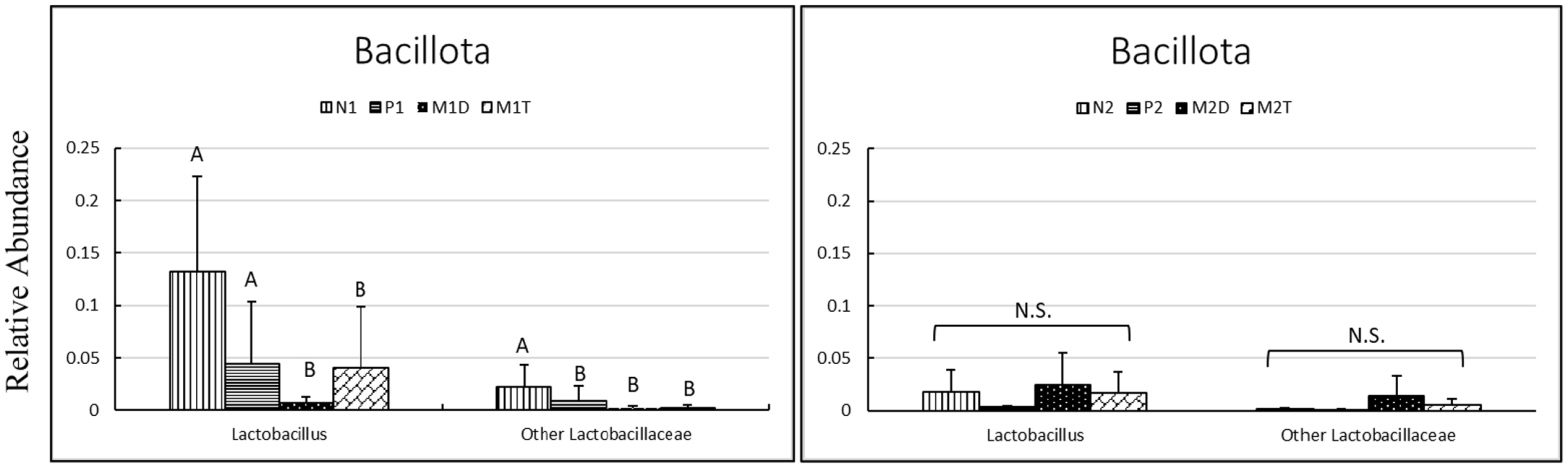
Relative abundance of the genus *Lactobacillus* during the study. Significant differences (*p* < 0.05) are indicated by different letters. N.S. indicates no significant difference.

**Figure 10:**
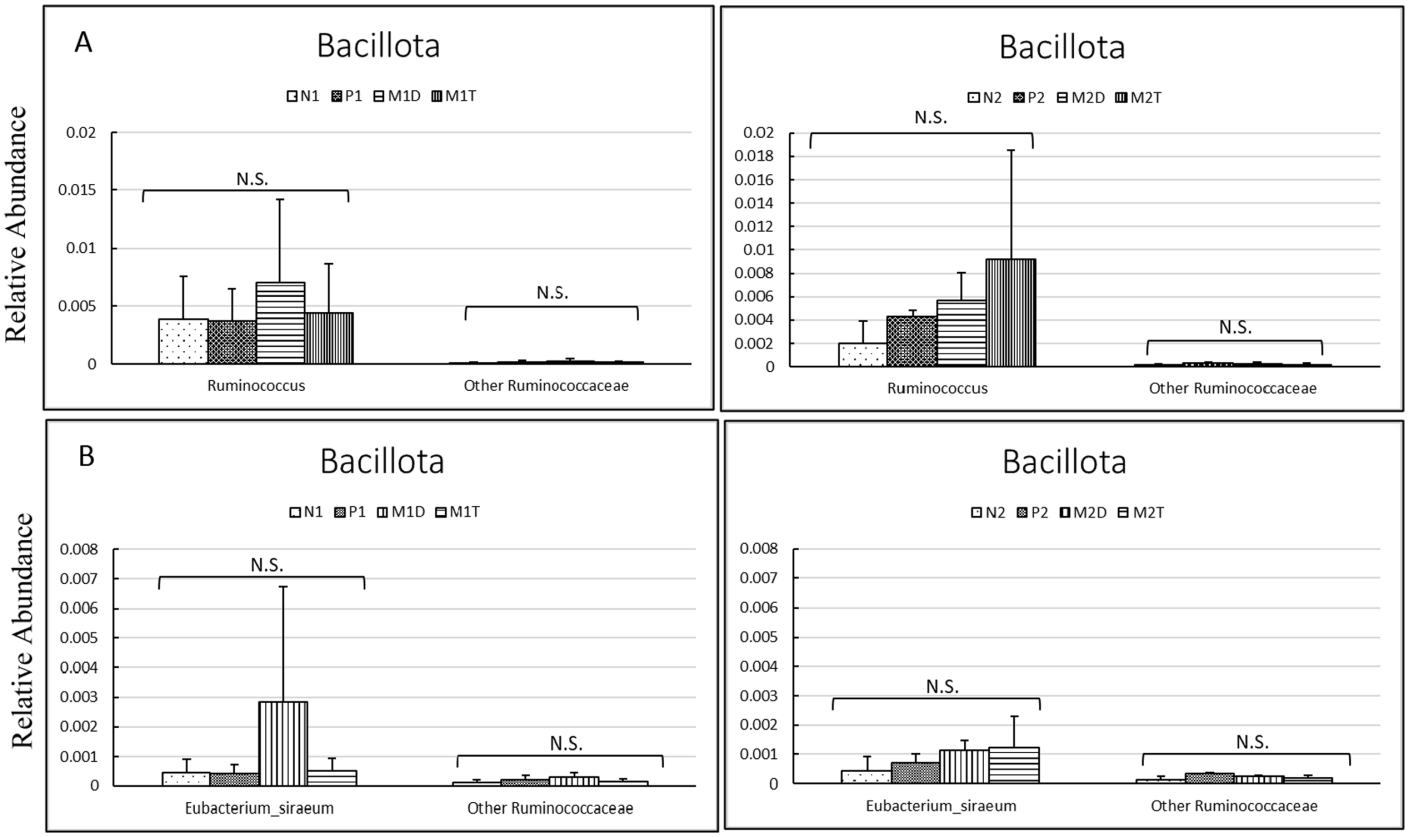
Relative abundance of Ruminococcaceae’s genera during the study: **(A)** *Ruminococcus*; **(B)** *Eubacterium_siraeum*. Significant differences (*p* < 0.05) are indicated by different letters. N.S. indicates no significant difference.

**Figure 11:**
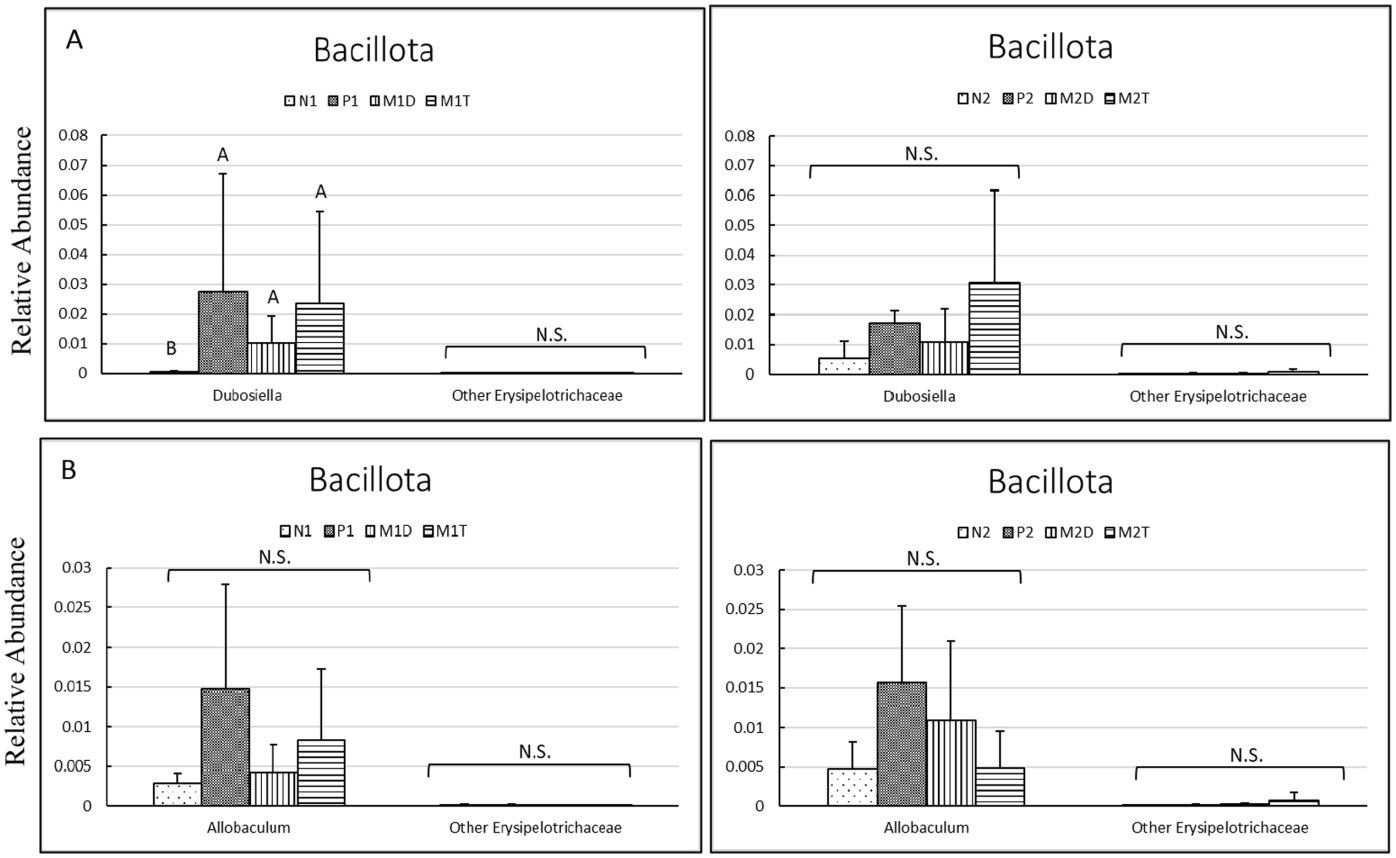
Relative abundance of Erysipelotrichaceae’s genera during the study. **(A)** *Dubosiella*; **(B)** *Allobaculum*. Significant differences (*p* < 0.05) are indicated by different letters. N.S. indicates no significant difference.

The phylum Bacillota comprises several families, including Lachnospiraceae, Lactobacillaceae, Ruminococcaceae, Erysipelotrichaceae, and others. Several genera within Lachnospiraceae were altered following PhIP treatment. Notably, the relative abundance of *Roseburia*, a well-known butyrate-producing genus, slightly reduced in the M1D and M1T (PhIP-treated) groups compared to the N1 and P1 groups in the first month; however, no statistically significant differences were observed among the groups in the second month. In contrast, the relative abundance of *Blautia* showed a generally non-significant increase in the M1D and M1T groups compared to N1 and P1 in the first month but was significantly elevated in the M2D and M2T groups relative to N2 and P2 in the second month. Furthermore, the abundance of *Lachnospiraceae-*NK4A136 group was higher in the M1D and M1T groups than in N1 and P1 in the first month and remained elevated in the M2D and M2T groups compared to N2 and P2 in the second month (Figure 8). The relative abundance of the genus *Lactobacillus* significantly decreased in the M1D and M1T groups compared to the N1 and P1 groups in the first month, whereas no significant differences were observed among the groups in the second month (Figure 9). Within the family Ruminococcaceae, the genera affected were *Ruminococcus* and *Eubacterium siraeum*. The abundances of *Ruminococcus* and *Eubacterium siraeum* slightly increased in the M1D and M1T groups compared to N1 and P1 in the first month and further increased in the M2D and M2T groups compared to N2 and P2 in the second month (Figure 10). Within the family Erysipelotrichaceae, the genera *Dubosiella* and *Allobaculum* were influenced by the treatment. The M1D and M1T groups exhibited a pronounced increase in *Dubosiella*, which was maintained in the M2D and M2T groups. In addition, the abundance of *Allobaculum* increased in the M1D and M1T groups and remained elevated in the M2D and M2T groups (Figure 11).

**Figure 12:**
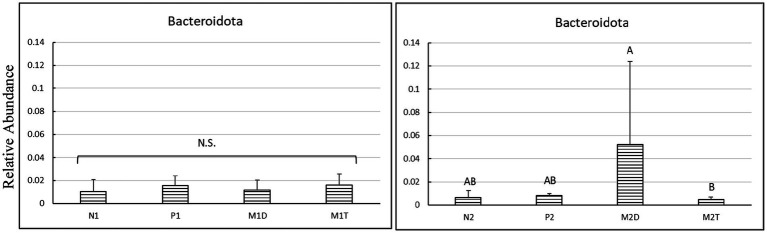
Relative abundance of the genus *Bacteroides* during the study. Significant differences (*p* < 0.05) are indicated by different letters. N.S. indicates no significant difference.

**Figure 13:**
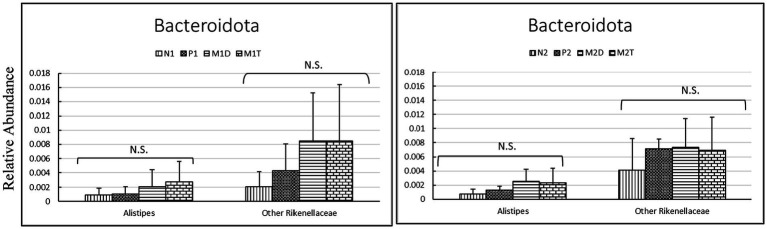
Relative abundance of the genus *Alistipes* during the study. N.S. indicates no significant difference.

**Figure 14:**
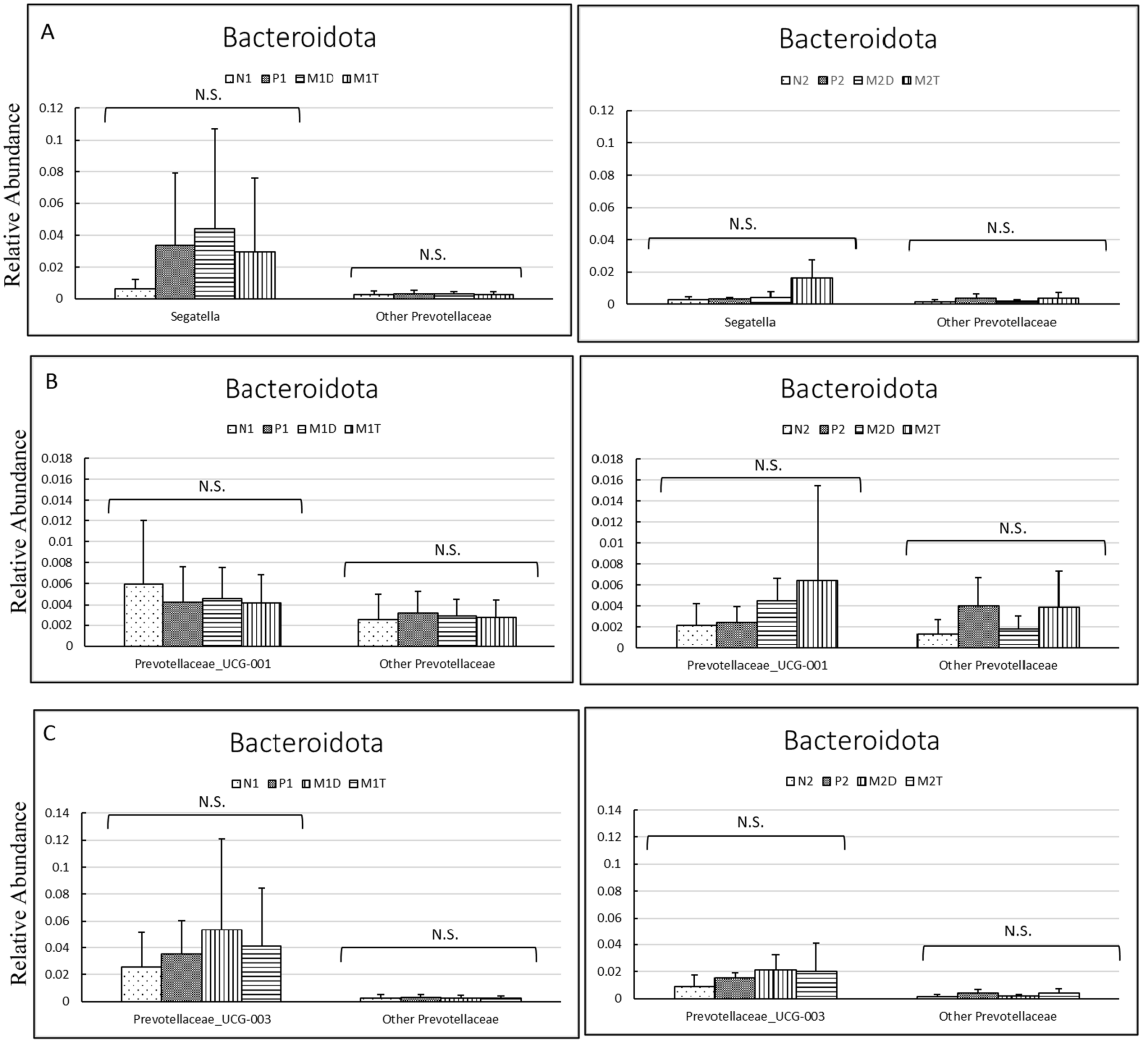
Relative abundance of Prevotellaceae’s genera during the study: **(A)** *Segatella*; **(B)** *Prevotellaceae_UCG-001*; **(C)** *Prevotellaceae_UCG-003*. N.S. indicates no significant difference.

**Figure 15:**
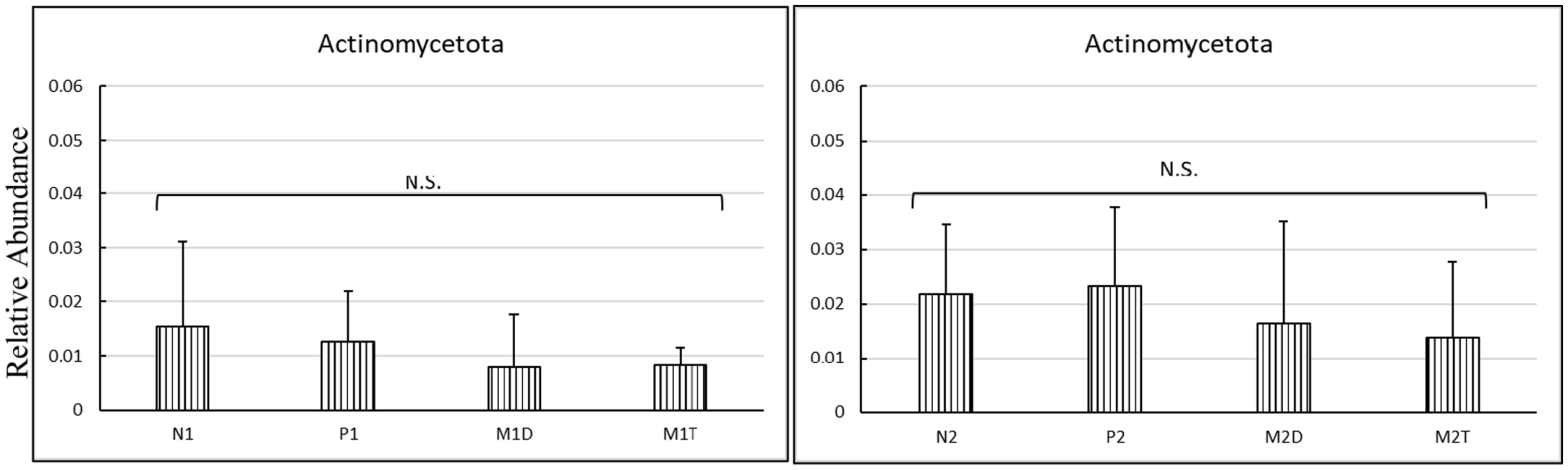
Relative abundance of the genus *Bifidobacterium* during the study. N.S. indicates no significant difference.

**Figure 16:**
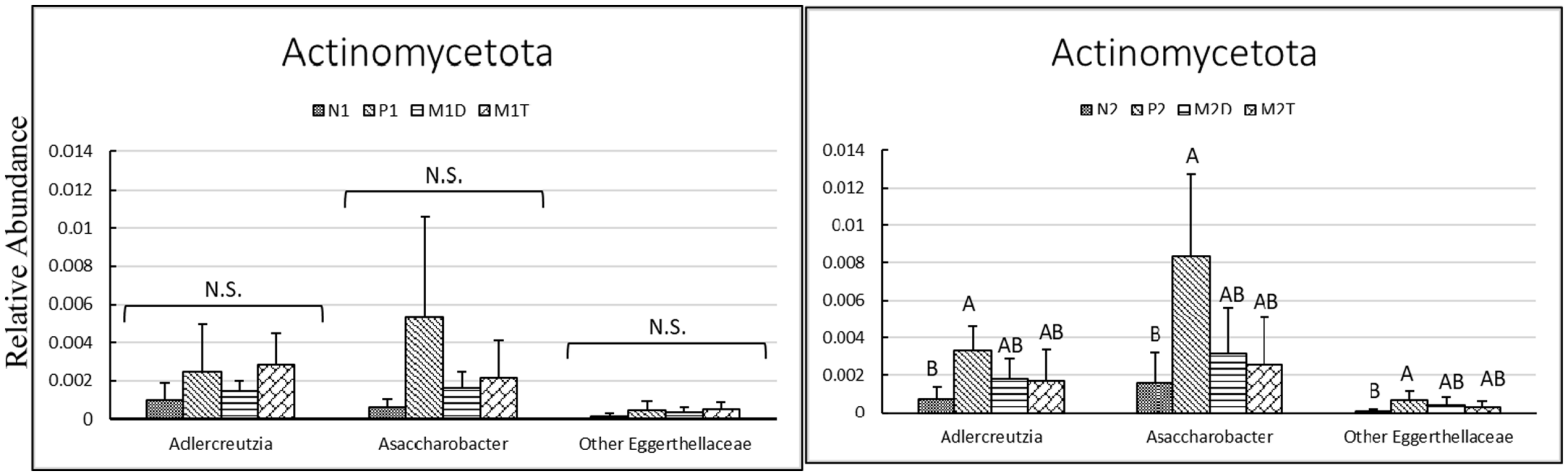
Relative abundance of Eggerthellaceae’s genera during the study. Significant differences (*p* < 0.05) are indicated by different letters. N.S. indicates no significant difference.

**Figure 17:**
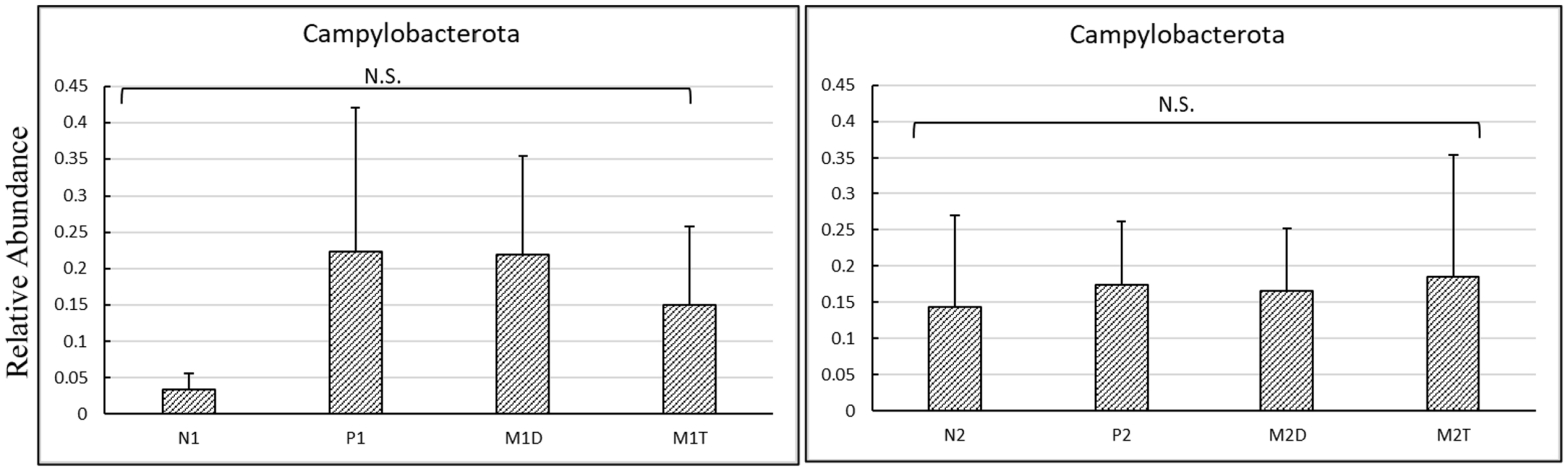
Relative abundance of the genus *Helicobacter* during the study. N.S. indicates no significant difference.

**Figure 18:**
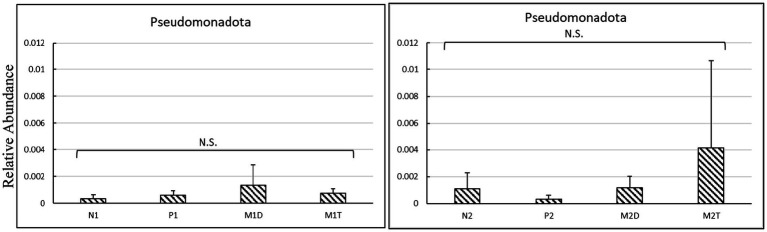
Relative abundance of the genus *Escherichia–Shigella* during the study. N.S. indicates no significant difference.

Several genera within the phylum Bacteroidota were significantly affected in this study. The relative abundance of the genus *Bacteroides* did not differ significantly among the experimental groups in the first month; however, a significant increase was observed in the M2D group compared to the other groups in the second month (Figure 12). The genus *Alistipes* exhibited increased relative abundance in the M1D and M1T groups compared to the N1 and P1 groups in the first month, and its abundance was likewise elevated in the M2D and M2T groups compared to the N2 and P2 groups in the second month (Figure 13). Although the abundances of *Segatella* and *Prevotellaceae*_UCG-003 did not differ significantly among the groups in either the first or second month, a trend toward increased abundance was observed in the treated groups over the course of the study. The relative abundance of *Prevotellaceae*_UCG-001 was slightly higher in the N1 group compared to the other groups in the first month, whereas an increase was detected in the treated M2D and M2T groups relative to N2 and P2 in the second month (Figures 14A–C). Within the phylum Actinomycetota, the dominant genus was *Bifidobacterium*, which showed higher relative abundance in the negative and positive control groups compared to the treated groups throughout the study, although these differences were not statistically significant in either the first or second month (Figure 15). In addition, the genera *Adlercreutzia* and *Asaccharobacter* exhibited increased relative abundance in the M1D and M1T groups in the first month and were significantly increased in the M2D and M2T groups in the second month (Figure 16). Within the phylum Campylobacterota, the genus *Helicobacter* showed a decrease in relative abundance in the N1 group compared to the treated M1D and M1T groups in the first month; however, no significant differences among the groups were detected in the second month (Figure 17). Among the Proteobacteria, the responsive genus was *Escherichia*–*Shigella*, which did not differ significantly among the groups in the first month but exhibited a marked increase in the M2T group in the second month (Figure 18).

## 4 Discussion

PhIP is formed at elevated temperatures during the cooking of meat, particularly through high-heat methods such as grilling, pan-frying, and barbecuing, and its formation is especially pronounced when cooking temperatures reach approximately 150–300 °C (Felton et al., 1986). It has been documented that PhIP is initially metabolized in the liver by two enzymes, specifically cytochrome P450 and CYP1A2 (Nakagama et al., 2005). In addition, a previous study demonstrated that exposure to PhIP induced perturbations in the composition of the intestinal microbiota (Zhao et al., 2020). Previous studies have demonstrated that the human gut microbiota has the capacity to biotransform PhIP into a conjugated metabolite, designated PhIP-M1. Specifically, it has been shown that the intestinal bacterium *Eubacterium hallii* can efficiently catalyze the conversion of PhIP to PhIP-M1 (Fekry et al., 2016). Accordingly, the objective of this study was to determine whether a two-month exposure to PhIP induces alterations in the composition of the cecal microbiota in mice. In this study, it was found that the distribution of OTUs varied between the negative control group and the treated group (PhIP), which is consistent with an earlier study that reported that the Venn diagram showed differences in OTU distribution between the CK (control) and PhIP groups. A total of 1,070 OTUs were shared by the two groups, while 447 and 514 unique OTUs were identified in the CK and PhIP groups, respectively (Zhao et al., 2020).

Richness and evenness constitute the principal components of bacterial α-diversity and serve as indicators of ecological resilience and stability. In the present study, the Chao1, observed species, and Shannon indices revealed reduced species richness, evenness, and distribution in the PhIP-treated groups relative to the negative control group. These findings align with previous reports documenting decreases in species richness and evenness following PhIP exposure (Zhao et al., 2021). Furthermore, a previous study reported that the CK (control) group exhibited greater microbial richness and evenness than the PhIP-treated group, as reflected by the Shannon, Simpson, Sobs, ACE, and Chao1 diversity indices (Zhao et al., 2020). In this study, the predominant bacterial phyla were Bacillota, Bacteroidota, Campylobacterota, Actinomycetota, and Pseudomonadota. Relative to the negative control group, the PhIP-treated group showed a reduced relative abundance of Bacillota and increased relative abundances of Bacteroidota and Campylobacterota. These findings are consistent with a previous report indicating that PhIP exposure disrupts gut microbiota composition and decreases the relative abundance of Firmicutes (Zhao et al., 2021). Moreover, a previous study found that PhIP caused a significant reduction in members of the Ruminococcaceae and Lactobacillaceae families (Zhao et al., 2021). In this study, it was found that the genera *Roseburia* and *Lactobacillus*, which are considered probiotic bacteria that support the GIT and immune system, slightly decreased in the PhIP-treated group compared to the negative control group. In addition, PhIP exposure has been reported to induce shifts in the composition of the gut microbiota at the genus level, characterized by reduced relative abundances of *Lactobacillus*, *Ruminococcus*_2, and *Ruminococcus*_1 (Zhao et al., 2021).

It was also reported that multiple bacterial taxa previously linked to proinflammatory responses, including *Bacteroides massiliensis*, *Lachnospira*, *Ruminococcus*, and *Eubacterium fissicatena*, were associated with intestinal inflammation (Liu et al., 2025). In addition, elevated relative abundance of *Eubacterium siraeum* may be indicative of intestinal dysbiosis, a condition that can adversely affect overall health status and gastrointestinal motility (Lai et al., 2024). Moreover, *Ruminococcus gnavus* has been reported to be associated with a growing range of intestinal and extraintestinal diseases, from inflammatory bowel disease (IBD) to neurological disorders (Crost et al., 2023). In addition, a previous study reported that certain species of *Allobaculum mucolyticum* are associated with inflammatory bowel disease (IBD) (van Muijlwijk et al., 2021). Moreover, *Blautia* and *Lachnospiraceae_NK4A136* have been reported to play a role in reducing the risk of metabolic diseases (Pecyna et al., 2025; Wu et al., 2020). In this study, we observed increased relative abundances of *Blautia*, *Lachnospiraceae*_NK4A136, *Ruminococcus*, *Eubacterium siraeum*, *Dubosiella*, and *Allobaculum* in the PhIP-treated groups compared to the negative control group.

Previous reports have indicated that certain species, such as *Bacteroides fragilis*, function as opportunistic human pathogens and are capable of inducing infections within the peritoneal cavity (Sears, 2001). A previous study reported that *Alistipes* spp. are strongly associated with dysbiosis and have been implicated in the pathogenesis of colorectal cancer (Parker et al., 2020). In addition, a previous study found that the highest abundance of *Alistipes* was present in stool samples from a Crohn’s disease-like ileitis mouse model, indicating a positive association between *Alistipes* and colitis (Rodriguez-Palacios et al., 2018). In this study, a marked increase in the relative abundance of the genus *Bacteroides* spp. was observed in the M2D (PhIP)-treated group. In addition, the genus *Alistipes* exhibited a modest increase in relative abundance in the PhIP-treated group. These observations are consistent with previous reports indicating elevated proportions of *Alistipes* spp. and *Bacteroides* spp. in the PhIP + DSS treatment group, as well as in groups receiving dietary fiber supplementation (Zapico et al., 2024). Elevated abundances of *Alistipes* spp. and *Bacteroides* spp. have been documented in the context of high-fat dietary regimens, which are linked to increased production of lipopolysaccharides and reactive oxygen species—molecular mediators that may contribute to the promotion of tumorigenesis (Liu et al., 2025). These findings contrast with those of a previous study, which reported that the relative abundances of *Alistipes* spp. and *Bacteroides* spp. decreased in response to the consumption of processed fermented meat (Lee et al., 2024).

In this study, we observed that the relative abundances of several genera within the family Prevotellaceae, including *Segatella*, *Prevotellaceae*_UCG-001, and *Prevotellaceae*_UCG-003, increased in the PhIP-treated groups compared to the negative control group. Consistent with these findings, a previous study reported an elevated relative abundance of *Prevotellaceae_UCG-001* in the PhIP-exposed group in an analysis of murine fecal samples (Zhao et al., 2021). *Prevotellaceae_UCG-001* is a key commensal bacterium of the gastrointestinal tract that contributes to the degradation of dietary substrates but can function as an opportunistic pathogen under specific conditions (Yang et al., 2024). It has also been reported that *Segatella* species, formerly known as *Prevotella copri*, have been linked to several health problems, including rheumatoid arthritis, low-grade systemic inflammation in HIV infection, and glucose intolerance (Xiao et al., 2024). *Bifidobacterium* spp., a genus of bacteria commonly recognized for its probiotic properties, exhibited a slight decrease in relative abundance in the PhIP-treated groups compared to the control group. This observation is consistent with previous findings indicating that PhIP intake reduces the relative abundance of *Bifidobacterium* spp. (Zhao et al., 2021). Furthermore, it has been demonstrated that members of the genera *Adlercreutzia* and *Asaccharobacter* possess the metabolic capacity to biotransform dietary isoflavones, abundant in numerous plant species, particularly soybeans, into equol (4′,7-isoflavandiol) (Singh et al., 2023; Wang et al., 2005; Vázquez et al., 2017). In this study, PhIP administration led to a progressive increase in the relative abundance of *Adlercreutzia* and *Asaccharobacter* compared to the control group, indicating that these taxa may contribute substantially to the metabolic processing of dietary components. *Helicobacter* spp., which are implicated in gastric carcinogenesis, are capable of colonizing the stomach owing to their ability to withstand highly acidic conditions. It has been demonstrated that DNA damage induced by the heterocyclic amines MeIQx and PhIP is greater in *H. pylori-*infected groups than in non-infected groups (Lochhead and El-Omar, 2007). In the present study, an increased abundance of *Helicobacter* spp. was observed in the PhIP-treated group compared to the control group. *Escherichia–Shigella* encompasses a cluster of closely related bacterial species within the family Enterobacteriaceae, among which *Shigella* represents a pathogenic subgroup of *Escherichia coli* responsible for shigellosis. Consistent with these findings, a previous investigation reported that the relative abundance of *Escherichia–Shigella* was elevated in mice fed a diet containing high levels of fried soybean oil (Hu et al., 2024). In this study, the relative abundance of the *Escherichia*–*Shigella* genus was elevated in the PhIP-treated group compared to the control group.

## 5 Conclusion

Overall, PhIP exposure could affect the cecal microbiota by decreasing the relative abundance of Bacillota and Actinomycetota and increasing the relative abundance of Bacteroidota and Campylobacterota. These findings suggest that treatment with PhIP changes the relative abundance and overall composition of cecal bacterial communities, leading to dysbiosis of the cecal microbiota after short-term exposure. However, it should be noted that there are several limitations, including a relatively small sample size, which might have limited the ability to produce strong statistical evidence for subtle differences, and a short exposure period, which may not have been sufficient to reveal long-term effects. Further research is thus warranted to examine the effects of chronic exposure to PhIP and to utilize a functional approach, such as combining 16S rRNA-based microbiota profiling with metagenomic sequencing.

## Data Availability

The raw data generated in this study can be found in the NCBI BioProject repository under accession PRJNA1468591.
