# Supplementary material for: Exposure to 2-amino-1-methyl-6-phenylimidazo[4,5-b]pyridine causes cecal microbiota dysbiosis in mice
Source: Front Microbiol. 2026 Jun 15;17:1859419. doi: 10.3389/fmicb.2026.1859419 (PMC13312906; doi:10.3389/fmicb.2026.1859419)
Supplement: Supplementary file 1 [file Data_Sheet_1.pdf]

Accession Sample Name SPUID Organism Tax ID BioProject SAMN60295664 N1C1 N1C1 mouse gut metagenome 410661 PRJNA1468591 SAMN60295665 N1C2 N1C2 mouse gut metagenome 410661 PRJNA1468591 SAMN60295666 N1C3 N1C3 mouse gut metagenome 410661 PRJNA1468591 SAMN60295667 N1C4 N1C4 mouse gut metagenome 410661 PRJNA1468591 SAMN60295668 P1C1 P1C1 mouse gut metagenome 410661 PRJNA1468591 SAMN60295669 P1C2 P1C2 mouse gut metagenome 410661 PRJNA1468591 SAMN60295670 P1C3 P1C3 mouse gut metagenome 410661 PRJNA1468591 SAMN60295671 P1C4 P1C4 mouse gut metagenome 410661 PRJNA1468591 SAMN60295672 M1D1 M1D1 mouse gut metagenome 410661 PRJNA1468591 SAMN60295673 M1D2 M1D2 mouse gut metagenome 410661 PRJNA1468591 SAMN60295674 M1D3 M1D3 mouse gut metagenome 410661 PRJNA1468591 SAMN60295675 M1D4 M1D4 mouse gut metagenome 410661 PRJNA1468591 SAMN60295676 M1T1 M1T1 mouse gut metagenome 410661 PRJNA1468591 SAMN60295677 M1T2 M1T2 mouse gut metagenome 410661 PRJNA1468591 SAMN60295678 M1T3 M1T3 mouse gut metagenome 410661 PRJNA1468591 SAMN60295679 M1T4 M1T4 mouse gut metagenome 410661 PRJNA1468591 SAMN60295680 N2C1 N2C1 mouse gut metagenome 410661 PRJNA1468591 SAMN60295681 N2C2 N2C2 mouse gut metagenome 410661 PRJNA1468591 SAMN60295682 N2C3 N2C3 mouse gut metagenome 410661 PRJNA1468591 SAMN60295683 N2C4 N2C4 mouse gut metagenome 410661 PRJNA1468591 SAMN60295684 P2C1 P2C1 mouse gut metagenome 410661 PRJNA1468591 SAMN60295685 P2C2 P2C2 mouse gut metagenome 410661 PRJNA1468591 SAMN60295686 P2C3 P2C3 mouse gut metagenome 410661 PRJNA1468591 SAMN60295687 P2C4 P2C4 mouse gut metagenome 410661 PRJNA1468591 SAMN60295688 M2D1 M2D1 mouse gut metagenome 410661 PRJNA1468591 SAMN60295689 M2D2 M2D2 mouse gut metagenome 410661 PRJNA1468591 SAMN60295690 M2D3 M2D3 mouse gut metagenome 410661 PRJNA1468591 SAMN60295691 M2D4 M2D4 mouse gut metagenome 410661 PRJNA1468591 SAMN60295692 M2T1 M2T1 mouse gut metagenome 410661 PRJNA1468591 SAMN60295693 M2T2 M2T2 mouse gut metagenome 410661 PRJNA1468591 SAMN60295694 M2T3 M2T3 mouse gut metagenome 410661 PRJNA1468591 SAMN60295695 M2T4 M2T4 mouse gut metagenome 410661 PRJNA1468591
